# Outcomes of Endovascular Treatment in Patients With Vertebrobasilar Artery Occlusion Beyond 24 Hours

**DOI:** 10.1001/jamanetworkopen.2025.15526

**Published:** 2025-06-13

**Authors:** Shuai Liu, Yongbo Xu, Thanh N. Nguyen, Feng Gao, Yuanyuan Xue, Shuling Liu, Sifei Wang, Bohao Zhang, Leilei Luo, Xuanye Yue, Binge Chang, Hong Li, Guodong Xu, Pinyuan Zhang, Yongchang Liu, Yibin Cao, Wanchao Shi, Shouchun Wang, Lile Zhao, Xiaoguang Tong, Ming Wei

**Affiliations:** 1 Huanhu Hospital Affiliated to Tianjin Medical University, Tianjin Medical University, Tianjin, China; 2 Department of Neurology, Boston University School of Medicine, Boston, Massachusetts; 3 Department of Radiology, Boston Medical Center, Boston, Massachusetts; 4 Department of Interventional Neuroradiology, Beijing Tiantan Hospital, Capital Medical University, Beijing, China; 5 Department of Neurosurgery, Tianjin Huanhu Hospital, Tianjin, China; 6 Department of Neurology, Tianjin Medical University General Hospital, China; 7 Department of Neurology, Tianjin First Central Hospital, China; 8 Department of Neurology, The Second Hospital of Tianjin Medical University, China; 9 Department of Neurology, Hebei General Hospital, Hebei, China; 10 Department of Neurosurgery, The Third Hospital of Hebei Medical University, China; 11 Department of Neurosurgery, Cangzhou City Central Hospital, Hebei Province, China; 12 Department of Neurosurgery, Tangshan Workers’ Hospital, Hebei Province, China; 13 Department of Neurology, Peking University BinHai Hospital, China; 14 Department of Neurology, The First Bethune Hospital of Jilin University, China; 15 Department of Neurology, Tianjin Xiqing Hospital, Tianjin, China

## Abstract

**Importance:**

The efficacy and safety of endovascular thrombectomy (EVT) plus best medical treatment (BMT) for vertebrobasilar artery occlusion beyond 24 hours remain uncertain.

**Objective:**

To evaluate outcomes associated with EVT in patients treated beyond 24 hours after last known well time due to vertebrobasilar artery occlusion.

**Design, Setting, and Participants:**

This multicenter, prospective cohort study enrolled patients between 2019 and 2024 from 11 comprehensive stroke centers across China. Eligible patients with vertebrobasilar artery occlusions treated beyond 24 hours after the estimated onset were included.

**Exposures:**

Patients were categorized into 2 groups: those who underwent EVT plus BMT and those who received BMT alone.

**Main Outcomes and Measures:**

The primary outcome was good functional status (modified Rankin Scale score, 0-3) at 90 days. Safety outcomes included symptomatic intracranial hemorrhage within 24 hours and 90-day mortality.

**Results:**

Among 202 patients with vertebrobasilar occlusion (158 male [78.2%]; median [IQR] age, 64.0 [56.2-70.0] years), 101 patients received EVT plus BMT and 101 patients received only BMT. The median (IQR) posterior circulation Acute Stroke Prognosis Early Computed Tomography Score was 8 (8-9), and the median (IQR) of time of onset to admission was 48 (24-96) hours. In the primary analysis using propensity score matching, 71 patients with EVT plus BMT had a higher rate of a good functional outcome at 90 days compared with 71 patients receiving BMT alone (41 patients [57.7%] vs 32 patients [45.1%]; adjusted risk ratio [aRR], 1.35 [95% CI, 1.02-1.79]). EVT plus BMT compared with BMT alone showed lower mortality (9 patients [12.7%] vs 20 patients [28.2%]; aRR, 0.27 [95% CI, 0.08-0.81]); differences in rates of symptomatic intracranial hemorrhage were not statistically significant (4 patients [5.6%] vs 0 patients; *P* = .13). A similar advantage in functional outcome for EVT plus BMT (aRR, 1.33 [95% CI, 1.04-1.71]) was observed in the inverse probability of treatment weighting analysis.

**Conclusions and Relevance:**

In this study, EVT plus BMT was associated with improved functional outcomes and survival rates at 90 days and a nonsignificant but numerically higher frequency of symptomatic intracranial hemorrhage than BMT alone in patients treated beyond 24 hours after last known well time. These findings suggest that randomized clinical trials comparing EVT with BMT in patients with acute vertebrobasilar artery occlusion are warranted.

## Key Points

**Question:**

What are the outcomes among patients undergoing endovascular thrombectomy for vertebrobasilar artery occlusion beyond 24 hours from the patient’s last known well time?

**Findings:**

In this cohort study of 202 patients with vertebrobasilar artery occlusion treated beyond 24 hours after last known well time, endovascular thrombectomy with best medical treatment was associated with a higher rate of good functional outcome at 90 days and lower mortality compared with best medical treatment alone.

**Meaning:**

These findings suggest that endovascular thrombectomy may be associated with improved functional outcomes in patients with vertebrobasilar artery occlusion presenting beyond 24 hours, underscoring the need for randomized clinical trials to evaluate the treatment’s effectiveness.

## Teaser

This cohort study investigates outcomes associated with endovascular treatment beyond 24 hours after last known well time among patients with vertebrobasilar artery occlusion.

## Introduction

Vertebrobasilar artery occlusion (VBAO) accounts for approximately 1% of all ischemic strokes and 5% of large vessel occlusion (LVO).^1,2,3,4,5^ Up to 80% of patients with VBAO experience severe disability and mortality.^6,7,8,9^ Results from observational studies and meta-analyses have been inconsistent regarding the association of EVT with improved clinical outcomes for patients with VBAO.^10,11,12,13,14^ After initial unsuccessful attempts,^8,15,16^ 2 randomized clinical trials (RCTs), the 2022 Endovascular Treatment for Acute Basilar-Artery Occlusion (ATTENTION) trial^9^ and the 2022 Trial of Thrombectomy 6 to 24 Hours After Stroke Due to Basilar-Artery Occlusion (BAOCHE),^17^ demonstrated clinical benefits of EVT for VBAO.^15^ These findings were subsequently validated by meta-analyses.^12^ A 2024 trial^18^ found that EVT in patients with low- to moderate-severity symptoms due to VBAO was not associated with an improved rate of favorable outcomes at 3 months. These trials demonstrated clinical benefits associated with EVT in VBAO, although the studies exclusively enrolled patients with acute ischemic stroke due to VBAO within 24 hours of symptom onset.

Although previous observational studies have suggested a potential benefit associated with EVT in patients with acute ischemic stroke beyond the 24-hour time window, these studies included anterior and posterior circulation occlusion with limited sample sizes, thus providing insufficient evidence.^19,20,21,22^ To our knowledge, no RCTs have assessed the efficacy and safety of EVT vs BMT in patients with VBAO beyond 24 hours. To address this critical evidence gap, our prospective study compared clinical outcomes between patients with VBAO who underwent EVT plus BMT vs those who underwent BMT alone beyond 24 hours after the last known well time.

## Methods

### Study Design and Population

This ongoing, prospective multicenter registry cohort study in China recruited eligible patients from 11 comprehensive stroke centers between January 2019 and February 2024. The study was reported in accordance with the Reporting of Studies Conducted using Observational Routinely Collected Health Data (RECORD) guideline.^23^ and the Strengthening the Reporting of Observational Studies in Epidemiology (STROBE) reporting guideline. The study was registered as the Triage of Patients Presenting Beyond 24 Hours With Ischemic Stroke Due to Vertebrobasilar Artery Occlusion (VBAO-Late) registry (NCT06510634). All participating centers followed the Expert Consensus on Standardized Diagnosis and Treatment in China, ensuring consistent data acquisition across sites and were certified comprehensive stroke centers, each performing more than 100 endovascular procedures annually. The interventional neuroradiologists at these centers had experience with 100 or more procedures. Data collected from each center were centrally consolidated at Tianjin Huanhu Hospital. The registry study was overseen by a steering committee, which was responsible for protocol implementation and monitoring of data collection. Ethics approval was obtained from the ethics committee of Tianjin Huanhu Hospital. Informed consent was provided by all patients or their legally authorized representatives before participation in the study.

Consecutive patients treated with EVT plus BMT or BMT alone were recruited from January 10, 2019, to February 8, 2024. The following inclusion criteria were applied: (1) aged 18 years or older; (2) basilar artery occlusion with or without intracranial vertebral artery occlusion, as determined on computed tomography (CT) angiography, magnetic resonance angiography, or digital subtraction angiography; (3) onset of VBAO symptoms more than 24 hours from last known well time; (4) no or mild prestroke disability, defined as a modified Rankin Scale (mRS) score of 2 or less; and (5) written informed consent by the patient or their legally authorized representative. Patients were excluded in the case of (1) neuroimaging evidence of cerebral hemorrhage on presentation; (2) unilateral vertebral artery occlusion but preserved antegrade flow in the basilar artery; (3) diagnosis by CT angiography or magnetic resonance angiography indicating basilar artery occlusion but with subsequent digital subtraction angiography revealing antegrade flow in the basilar artery; (4) lack of follow-up information on outcomes at 90 days; and (5) serious, advanced, or terminal illness.

Patients with acute stroke were evaluated for eligibility before entering this registry study. The treatment modality, EVT plus BMT or BMT alone, was determined by senior interventional neurologists (F.G., S.W., L.L., and M.W.) at the local stroke center. The EVT plus BMT group included patients treated with stent retriever, aspiration, balloon angioplasty or stenting, intra-arterial thrombolysis, or a combination of these treatments. The BMT alone group received BMT (eg, antiplatelet drugs, anticoagulation, or a combination of these medical treatments), as described in the guidelines for the management of acute ischemic stroke.^24^

We prospectively collected information on demographic variables (age and sex), medical history (diabetes, atrial fibrillation, hyperlipidemia, coronary heart disease, hypertension, and transient ischemic attacks or stroke), clinical parameters (systolic and diastolic blood pressure on admission and National Institutes of Health Stroke Scale [NIHSS] score on admission), pretreatment imaging findings (CT posterior circulation Acute Stroke Prognosis Early Computed Tomography Score [pc-ASPECTS], CT Pons-Midbrain Index [PMI], magnetic resonance imaging [MRI] pc-ASPECTS, and MRI PMI), type of treatment, complications, and outcomes. We considered 2 categories of essential data for our analysis: (1) baseline covariates required for propensity score calculation and matching and (2) outcome variables for assessing treatment outcomes. In case of missing data or extreme or inconsistent values, centers were contacted and asked to verify and correct records as appropriate.

Symptom onset was defined as the time at which the patient was last known to be free from acute stroke symptoms excluding isolated vertigo. For patients with wake-up stroke or an unwitnessed time of stroke onset because of unconsciousness or inability to speak, the time of symptom onset was calculated from the time at which the patient was last seen to be well.

### Outcomes

The primary outcome was good functional outcome, defined by a 90-day (within 14 days less than or more than 90 days) mRS score of 0 to 3, which indicates moderate disability but independent ambulation. The mRS was assessed at the 90-day follow-up during a regularly scheduled clinical visit by a board-certified physician (Y.X.) or, if the patient was unable to attend, through structured telephone interviews using the validated mRS-Structured Interview^25,26^ conducted by a trained research nurse (S.L.) who did not know the modality of treatment for patients.

The secondary outcome was the distribution of mRS scores at 90 days (shift analysis), functional independence at 90 days (mRS score, 0-2), comparisons of mRS scores (0 or 1 vs 2-6; 0-4 vs 5 or 6). Safety outcomes included stroke-related mortality within 90 days and the incidence of symptomatic intracranial hemorrhage (sICH) during hospitalization according to the Third European Cooperative Acute Stroke Study (ECASS III) criteria^27^ and any intracranial hemorrhage.

### Statistical Analysis

Continuous variables were reported as mean (SD) for normally distributed data or median (IQR) for nonnormally distributed data. The normality of distributions was assessed using histograms and the Kolmogorov-Smirnov test. Categorical variables are presented as numbers with percentages. Comparisons of demographic and baseline characteristics were conducted using the 2-sample *t* test or Wilcoxon rank-sum test for continuous variables and the χ^2^ test or Fisher exact test for categorical variables, as appropriate. We did not impute for missing data.

To mitigate bias, we applied 2 statistical methods to adjust for potential confounding factors. To balance baseline characteristics between the endovascular and control group, we used propensity score analysis to mitigate the effects of confounding factors and assess the robustness of our findings. Propensity scores were calculated using logistic regression based on the following characteristics: sex, age, admission blood pressure, hypertension, atrial fibrillation, smoking, hypertriglyceridemia, diabetes, baseline NIHSS score, estimated time from basilar artery occlusion to admission, occlusion sites (VBAO with vs without vertebral artery occlusion), baseline CT pc-ASPECTS, baseline CT PMI, baseline MRI pc-ASPECTS, and baseline MRI PMI. Initially, we matched patients who underwent EVT with those receiving BMT using propensity score matching (PSM) in a 1:1 ratio. Nearest-neighbor matching was used within a caliper width of 0.2 SDs of the logit of the propensity score without replacement. After PSM, *E*-values^28^ were calculated for variables with a standardized mean difference (SMD) between 0.1 and 0.2 to assess their potential association with the primary outcome and evaluate result robustness. We then performed inverse probability treatment weighting (IPTW) based on propensity scores. For the propensity score weighting population, the stabilized mean treatment effect weighting method was used to balance covariates while ensuring stable weights. Patients receiving EVT were assigned a weight using the following equation: proportion of patients receiving EVT)/(propensity score). Those receiving BMT were assigned a weight using the following equation: (1 − the proportion of patients receiving BMT)/(1 − propensity score). To enhance equipoise and minimize bias, tails of propensity score distributions were trimmed by excluding observations at or below the first percentile for EVT and at or above the 99th percentile for BMT. After propensity score analysis, binary outcomes were compared between EVT and BMT groups. In our primary analysis of the PSM cohort, the Poisson distribution with a log link function was used to estimate the relative risk, and the Gaussian distribution with an identity link function was used to estimate risk difference and mean difference. Ordinal outcomes, such as the distribution of mRS score at 3 months, were compared using ordinal logistical regression models. Data were inversely probability weighted and analyzed using the same Poisson regression model for primary and safety outcomes.

Subgroup analyses were conducted by propensity matching the data to evaluate by the following subgroups: age (18-74 years and ≥75 years), baseline NIHSS score (0-9 and 10-40), atrial fibrillation, baseline pc-ASPECTS (0-8 and >8), occlusion site (basilar artery and basilar with intracranial vertebral artery), and estimated time from basilar artery occlusion to admission (24-72 hours and >72 hours). Analysis was performed using logistic regression, including interaction terms, to assess the association between EVT and good functional outcome across subgroups.

All *P* values were 2-sided, with a significance level set at *P* < .05. Statistical analyses were performed using R statistical software version 4.2.3 (R Project for Statistical Computing).

## Results

From January 2019 to February 2024, a total of 225 patients were recruited across 11 comprehensive stroke centers (eFigure in Supplement 1). There was 1 center (with 8 patients) excluded from participation in the registry because not all pertinent data on consecutive patients were being recorded. Another 15 patients were excluded according to inclusion and exclusion criteria: 10 patients with unilateral vertebral artery occlusion but preserved antegrade flow in the basilar artery and 5 patients with basilar artery occlusion on magnetic resonance angiography but a patent basilar artery revealed with digital subtraction angiography. The final cohort comprised 202 patients (158 male [78.2%]; median [IQR] age, 64.0 [56.2-70.0] years) who completed follow-up, with outcome assessments conducted via structured telephone interviews among 155 patients (76.7%) and in-person clinical evaluations among 47 patients (23.3%). We excluded 6 patients with missing data required for the propensity score from matched analyses but included them in baseline analyses. Among all patients, the median (IQR) pc-ASPECTS was 8 (8-9) and the median (IQR) of time of onset to admission was 48 (24-96) hours. Therefore, 202 patients were categorized into 2 groups based on the treatment they received: 101 patients receiving EVT plus BMT and 101 patients receiving BMT alone.

Table 1 presents baseline characteristics of the study population before and after propensity score matching, showing balanced distribution of selected covariates after matching. In the propensity-matched cohort, 142 patients (71 patients in each group) were matched. The median (IQR) age in the BMT alone vs EVT plus BMT group was 62.5 (55.3-67) years vs 64.0 (55-69.8) years. There were 55 males (77.5%) in the EVT plus BMT group vs 56 males (78.9%) in the BMT alone group. The median (IQR) time from symptom onset to hospital admission was 49.0 (29.3-117.3) hours in the EVT plus BMT vs 48.0 (24.0-96.0) hours in the BMT alone group. The median (IQR) baseline NIHSS score was 12.0 (6.0-22.8) in the EVT plus BMT vs 10.0 (6.3-17.0) in the BMT alone group, and the median (IQR) CT pc-ASPECTS was 8 in both groups (EVT plus BMT: 8.0 (8.0-9.0]; BMT alone: 8.0 [7.5-9.0]).

**Table 1.** Baseline Patient Characteristics

Characteristic	Before matching				After matching			
	Patients, No. (%)		*P *value	SMD, %	Patients, No. (%)		*P *value	SMD, %
	BMT (n = 101)	EVT (n = 101)			BMT (n = 71)	EVT (n = 71)		
Age, median (IQR), y	63.0 (56.0-70.0)	64.0 (54.0-69.0)	.35	20.3	62.5 (55.25-67.0)	64.0 (55.0-69.8)	.70	3.9
Sex								
Male	77 (76.2)	81 (80.2)	.50	9.6	56 (78.9)	55 (77.5)	>.99	3.4
Female	24 (23.8)	20 (19.8)			15 (21.1)	16 (22.5)		
Blood pressure on admission, median (IQR), mm Hg								
Systolic	146.0 (136.0-158.0)	147.0 (130.0-165.0)	.77	2.0	147.0 (135.0-156.75)	146.5 (131.0-165.0)	.91	0.6
Diastolic	83.0 (77.0-90.0)	86.0 (77.0-91.0)	.89	11.5	84.0 (78.0-90.8)	85.0 (76.0-92.5)	.89	1.8
Baseline NIHSS score, median (IQR)	10.0 (7.0-17.0)	12.0 (6.0-22.0)	.34	18.5	10.0 (6.3-17.0)	12.0 (6.0-22.8)	.73	1.4
Medical history								
Atrial fibrillation	12 (11.9)	14 (13.9)	.674	<.001	9 (12.7)	10 (14.1)	.81	6.5
Hypertension	80 (79.2)	76 (75.3)	.50	9.5	56 (78.9)	56 (78.9)	>.99	<0.01
Hyperlipidemia	41 (40.6)	43 (42.6)	.89	4.0	29 (40.9)	30 (42.3)	1	2.9
Diabetes	36 (35.6)	36 (35.6)	>.99	<0.01	24 (33.8)	28 (39.4)	.60	11.7
Ischemic stroke or transient ischemic attack	21 (20.8)	27 (26.7)	.33	3.8	15 (21.3)	18 (25.4)	.55	6.3
Coronary heart disease	24 (23.8)	20 (19.8)	.50	22.7	14 (19.7)	11 (15.5)	.66	11.1
Smoking	48 (47.5)	34 (33.8)	.06	28.5	28 (39.4)	28 (39.4)	1	<0.01
Anticoagulant	19 (18.8)	15 (14.9)	.45	10.6	14 (19.7)	9 (12.7)	.36	19.2
Antiplatelet	20 (19.8)	16 (15.8)	.46	10.4	15 (21.1)	10 (14.1)	.38	18.6
OTA, median (IQR), h	48.0 (24.0-96.0)	48.0 (28.0-120.0)	.09	3.1	48.0 (24.0-96.0)	49.0 (29.3-117.3)	.51	15.1
Imaging score, median (IQR)^a^								
Baseline CT pc-ASPECTS	8.0 (8.0-10.0)	8.0 (7.0-9.0)	.18	14.7	8.0 (7.5-9.0)	8.0 (8.0-9.0)	.71	9.2
Baseline CT PMI	0.0 (0.0-1.0)	0.0 (0.0-1.0)	.29	19.1	0.0 (0.0-1.0)	0.0 (0.0-1.0)	.71	<0.01
Baseline MRI pc-ASPECTS	7.0 (6.0-8.0)	8.0 (6.0-9.0)	.76	3.6	7.0 (6.0-8.0)	8.0 (6.0-9.0)	.41	14.4
Baseline MRI PMI	1.0 (0.0-2.0)	1.0 (0.0-2.0)	.76	1.0	1.0 (0.0-2.0)	1.0 (0.0-2.0)	.23	14.0
Stroke causative mechanism								
Large artery atherosclerosis	87 (86.1)	84 (83.7)	>.99	<0.01	61 (85.9)	60 (84.5)	.93	6.5
Cardioembolism	12 (11.9)	14 (13.9)			9 (12.7)	10 (14.1)		
Other or unknown	2 (2.0)	3 (3.0)			1 (1.4)	1 (1.4)		
Occlusion site								
Basilar artery	34 (33.7)	53 (52.5)	.01	38.7	30 (42.3)	34 (47.9)	.61	11.3
Basilar artery and intracranial vertebral artery	67 (66.3)	48 (47.5)			41 (57.8)	37 (52.1)		
Transfer								
Direct admission	75 (74.3)	81 (80.2)	.31	14.2	50 (70.4)	60 (84.5)	.07	34.2
Transfer to hospital	26 (25.7)	20 (19.8)			21 (29.6)	11 (15.5)		

Abbreviations: BMT, best medical treatment; CT, computed tomography; EVT, endovascular treatment; MRI, magnetic resonance imaging; NIHSS, National Institutes of Health Stroke Scale; pc-ASPECTS, posterior circulation Acute Stroke Prognosis Early Computed Tomography Score; PMI, Pons Midbrain Index; OTA, estimated time from basilar artery occlusion to admission.

^a^ There were 2 patients (1 in the EVT group and 1 in the BMT group) who lacked CT pc-ASPECTS and PMI scores and 4 patients (1 in the EVT group and 3 in the BMT group) who lacked MRI pc-ASPECTS and PMI scores.

### Outcomes by Treatment Arm in PSM Cohort

**Figure 1.**
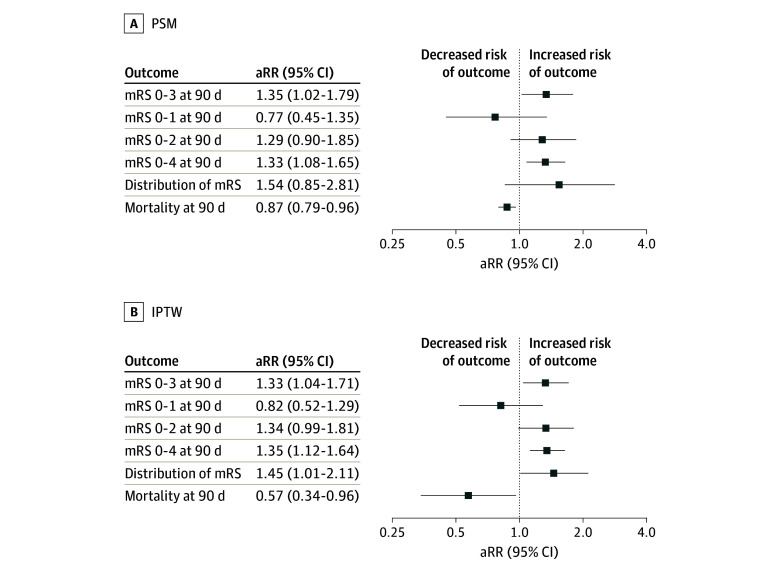
Adjusted Association of Endovascular Treatment With Clinical and Safety Outcomes A, Propensity score matching (PSM) cohort–based analysis and B, inverse probability of treatment weighting (IPTW) cohort–based analysis are presented. aRR indicates adjusted risk ratio; mRS, modified Rankin Scale.

**Figure 2.**
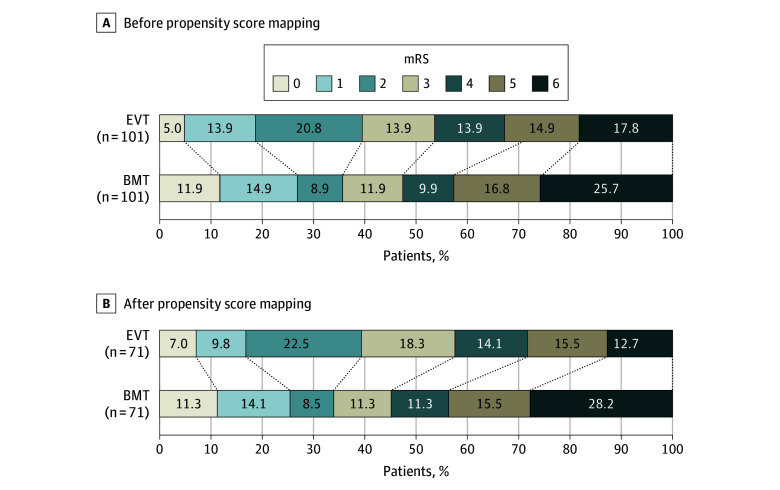
Modified Rankin Scale Score (mRS) Distribution The distribution is shown at 90 days A, before propensity score matching and B, after propensity score matching. Scores on the mRS range from 0 to 6, with a score of 0 indicating no symptoms, 1 no clinically significant disability, 2 slight disability (able to handle own affairs without assistance but unable to carry out all previous activities), 3 moderate disability necessitating some help but ability to walk unassisted, 4 moderately severe disability (unable to attend to bodily needs and unable to walk), 5 severe disability (receiving constant nursing care and attention), and 6 (death). BMT indicates, best medical treatment; EVT, endovascular treatment.

In the analysis using the PSM cohort, the primary outcome of a 90-day mRS score of 0 to 3 occurred more frequently in patients treated with EVT plus BMT than in patients treated with BMT alone (41 patients [57.7%] vs 32 patients [45.1%]; adjusted risk ratio [aRR], 1.35 [95% CI, 1.02-1.79]). (Table 2; Figure 1). The proportion of patients achieving functional independence was similar between EVT and BMT groups (90-day mRS score, 0-2: 28 patients [39.4%] vs 24 patients [33.8%]; aRR, 1.29 [95% CI, 0.90-1.85]). The distribution of 90-day mRS scores by treatment arm before and after PSM is shown in Figure 2. There was no difference in the distribution of the mRS score between EVT plus BMT and BMT alone groups, with an adjusted odds ratio (aOR) of 1.54 (95% CI, 0.85-2.81) (eTable 1 in Supplement 1).

**Table 2.** Outcomes by Treatment Group After Propensity Score Matching

Outcome	Patients, No. (%)		Adjusted effect size
	EVT (n = 71)	BMT (n = 71)	
Primary outcome: mRS score 0 to 3 at 90 d^a^	41 (57.7)	32 (45.1)	1.35 (1.02 to 1.79)^b^
Secondary outcomes			
mRS score at 90 d			
Median (IQR)^c^	3 (2 to 5)	4 (1.5 to 6)	1.54 (0.85 to 2.81)^d^
0 to 1	12 (16.9)	18 (25.4)	0.77 (0.45 to 1.35)^b^
0 to 2	28 (39.4)	24 (33.8)	1.29 (0.90 to 1.85)^b^
0 to 4	51 (71.8)	31 (43.7)	1.33 (1.08 to 1.65)^b^
Reperfusion on digital subtraction angiography^e^	58 (81.7)	NA	NA
NIHSS score at 5 to 7 d or discharge, median (IQR)^f^	8 (3 to 14)	10 (4 to 25)	−4.39 (−7.40 to −1.40)^g^
Safety outcomes			
Death within 90 d	9 (12.7)	20 (28.2)	0.27 (0.08 to 0.81)^b^
Symptomatic intracranial hemorrhage	4 (5.6)	0	.13^h^
Other serious adverse events			
Pneumonia	32 (45.1)	23 (32.4)	.12^h^
Malignant brain edema	8 (11.3)	7 (9.9)	.79^h^
Acute heart failure	10 (14.1)	13 (18.3)	.49^h^
Gastrointestinal hemorrhage	1 (1.5)	5 (7.04)	.21^h^
Acute respiratory failure	12 (16.9)	15 (21.1)	.52^h^

Abbreviation: BMT, best medical treatment; EVT, endovascular treatment; mRS, modified Rankin Scale; NA, not applicable; NIHSS, National Institutes of Health Stroke Scale.

^a^ An mRS score of 0 to 3 was defined as a favorable clinical outcome, using the modified Poisson regression model.

^b^ Adjusted risk ratio with 95% CI.

^c^ The mRS score at 90 days was assessed using ordinal logistic regression models.

^d^ Common odds ratio with 95% CI.

^e^ Reperfusion on digital subtraction angiography was defined as a modified thrombolysis in cerebral infarction grade of 2b or 3. A modified thrombolysis in cerebral infarction reperfusion grade of 2b or higher indicates antegrade reperfusion of more than half the ischemic territory of the previously occluded target artery.

^f^ There were 3 patients (1 in the EVT group and 2 in the BMT group) who lacked NIHSS scores (secondary outcome) at discharge.

^g^ Mean difference with 95% CI.

^h^ *P* value.

### Safety Outcomes

In the PSM cohort, the EVT plus BMT group had a numerically higher rate of symptomatic intracranial hemorrhage than the BMT alone group, although this difference was not statistically significant (4 patients [5.6%] vs 0 patients; *P* = .13). The 90-day mortality was lower in patients treated with EVT plus BMT than in those who received BMT alone (9 patients [12.7%] vs 20 patients [28.2%]; aRR, 0.27 [95% CI, 0.08-0.81]); the overall mortality in the PSM cohort was 29 patients (20.4%).

### Subgroup Analysis

**Figure 3.**
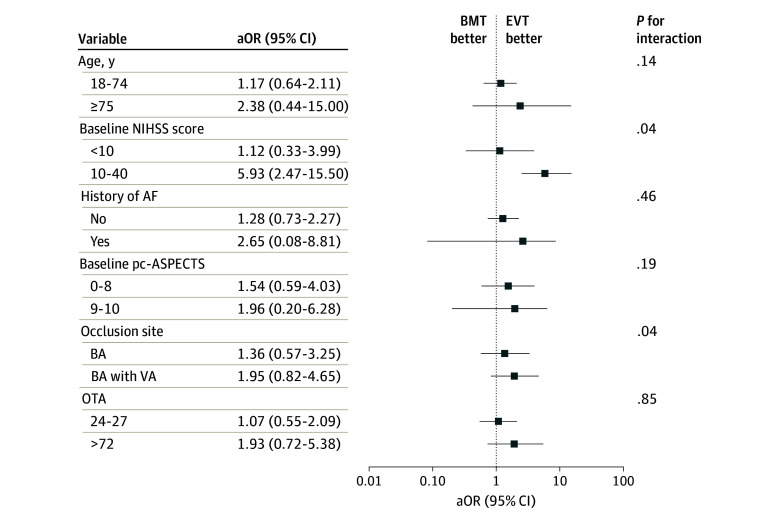
Subgroup Analyses of Clinical Outcomes in Propensity Score Matching Cohort AF indicates atrial fibrillation; aOR, adjusted odds ratio; BA, basilar artery; BMT, best medical treatment; EVT, endovascular treatment; NIHSS, National Institutes of Health Stroke Scale; OTA, estimated time from basilar artery occlusion to admission; pc-ASPECTS, posterior circulation Acute Stroke Prognosis Early Computed Tomography Score; VA, vertebral artery.

We performed subgroup analysis for the PSM cohort. The association between EVT plus BMT and the presence of a favorable clinical outcome at 90 days was consistent across subgroups, except for an interaction observed for baseline NIHSS score. The adjusted difference in the rate of an mRS score of 0 to 3 between EVT and BMT was significantly greater in patients with baseline NIHSS scores of 10 or greater in the PSM cohort (adjusted odds ratio [aOR], 5.93 [95% CI, 2.47-15.50]) compared with patients with baseline NIHSS scores less than 10 (aOR, 1.12 [95% CI, 0.33-3.99]; *P* for interaction = .04) (Figure 3).

### Sensitivity Analysis

Figure 1 summarizes results of the logistic regression analysis using IPTW. In our sensitivity analysis using the IPTW-based analysis, including 202 patients (101 patients in EVT plus BMT and 101 patients in BMT alone) after selection, EVT was associated with higher odds of achieving mRS scores of 0 to 3 at 90 days (aRR, 1.33 [95% CI, 1.04-1.71]). There was an improvement in the distribution of the mRS score, with a common OR of 1.45 (95% CI, 1.01-2.11) favoring EVT plus BMT. The 90-day mortality was lower in patients treated with EVT than in those who received BMT alone (aRR, 0.57 [95% CI, 0.34-0.96]).

We calculated *E*-values for potential confounders after matching. For the primary outcome, the calculated *E*-value was 3.7. Furthermore, *E*-value analyses were extended to covariates showing residual imbalances (SMD >0.1 to <0.2) after propensity score matching (eTable 2 in Supplement 1). Analysis of results by *E*-value revealed that variables with an SMD greater than 0.1 were not associated with the primary outcome.

## Discussion

Our multicenter cohort study revealed that among eligible patients with acute ischemic stroke due to VBAO who presented beyond 24 hours after symptom onset, EVT plus BMT was associated with an increased likelihood of good functional outcomes compared with BMT alone. Furthermore, patients receiving EVT plus BMT had a lower mortality rate compared with those receiving BMT alone, despite a higher incidence of sICH in the EVT plus BMT group.

The effectiveness of EVT for patients with VBAO who underwent EVT within 24 hours of symptom onset was established in prior studies. A 2023 meta-analysis^12^ of RCTs demonstrated that EVT for patients with VBAO who presented at less than 24 hours was associated with a higher likelihood of achieving a good functional outcome (OR 1.99 [95% CI, 1.04-3.80]; *P* = .04). Current American Heart Association guidelines, which have not yet been updated with recent trial data, recommend EVT for patients with VBAO within 6 hours of symptom onset (class IIb recommendation).^29^ In contrast, other societies further endorse EVT for patients with VBAO presenting between 12 and 24 hours under class IIa evidence.^30,31^ However, the role of EVT for patients with VBAO presenting beyond 24 hours remains controversial and necessitates urgent validation through RCTs.

A previous RCT^32^ investigating EVT for VBAO used an mRS score of 0 to 3 as the primary outcome. Aligning with this approach, our study also used an mRS score of 0 to 3 as the primary end point, with good functional outcomes achieved in 57.7% of patients with VBAO treated with EVT and 45.1% of those receiving BMT. However, direct comparisons with prior studies of VBAO EVT beyond 24 hours are constrained because most studies report only mRS score outcomes of 0 to 2. Notably, Pandhi et al^22^ observed that 1of 10 (10.0%) patients with VBAO treated beyond 24 hours achieved an mRS score of 0 to 2. Other studies have included patients with posterior circulation strokes in this very late window but analyzed them alongside anterior circulation cases, failing to providing isolated VBAO data.^19,20^

Comparing our results with earlier positive RCTs conducted within 24 hours (the BAOCHE^17^ and ATTENTION^9^ trials), we observed that while these studies demonstrated significant EVT benefits compared with BMT (mRS score, 0-3: 46%-46.4% vs 23%-24.3%), our cohort exhibited even higher rates of good functional outcomes. This discrepancy may be attributed to several reasons. First, our cohort had better imaging features compared with the BAOCHE population, as indicated by comparably reduced baseline median (IQR) CT PMI scores for EVT vs BMT groups in our study (0 [0-1] vs 0 [0-1]) and in BAOCHE (1 [0-2] vs 1 [0-2]). Second, the milder baseline neurological severity in our population, evidenced by lower baseline median (IQR) NIHSS scores in EVT vs BMT groups for our study (12 [6-22] vs 10 [7-17]) compared with BAOCHE (20 [15-29] vs 19 [12-30]) and ATTENTION (24 [15-35] vs 24 [14-35]), likely contributed to better prognoses in our study.

Regarding safety outcomes, our study found a higher incidence of sICH in the EVT group compared with BMT alone (4 patients [5.6%] vs 0 patients). This aligns with findings from Pandhi et al,^22^ who reported a 10% sICH rate in patients with VBAO treated with EVT beyond 24 hours. Notably, these rates remain comparable to those observed in positive RCTs conducted within 24 hours (sICH range, 5%-9% in BAOCHE^17^ and ATTENTION^9^). The comparable sICH rates between our cohort and previous studies suggest that delayed reperfusion, even beyond 24 hours, is not intrinsically associated with increased risk of sICH when patients are carefully selected. Importantly, the overall 90-day mortality rate in our study (20.4%) was substantially lower than in prior VBAO trials, such as BAOCHE (36%)^17^ and ATTENTION (43%),^9^ for which no data exist for patients with VBAO treated beyond 24 hours. Crucially, EVT was associated with reduced mortality compared with BMT alone in our study, highlighting its potential lifesaving benefit that may be observed in carefully selected patients presenting after 24 hours.

Subgroup analyses revealed no interaction between delayed treatment initiation and EVT effectiveness, indicating that time from symptom onset does not preclude better prognosis in carefully selected patients. This finding aligns with the Late Triage of Patients Presenting Beyond 24 Hours With Acute Ischemic Stroke Due to Large Vessel Occlusions (TRACK-LVO Late) study,^33^ which similarly demonstrated no treatment timing–effectiveness interaction in late-presenting patients with anterior circulation LVO. However, baseline neurological severity appears to be a key factor associated with EVT effectiveness. Patients with baseline NIHSS scores of 10 or greater demonstrated significantly better outcomes with EVT compared with BMT alone (aOR, 5.93 [95% CI, 2.47-15.50]), whereas those with milder deficits (NIHSS score <10: aOR, 1.12 [95% CI, 0.33-3.99]) did not. These findings align with results from the ATTENTION registry^34^ (aRR, 1.58 [95% CI, 1.30-1.91] for NIHSS scores ≥10) and BASICS trial^16^ (aRR, 1.45 [95% CI, 1.03-2.04] for NIHSS scores ≥10), collectively suggesting that baseline neurological severity may be a critical factor associated with EVT effectiveness in late-presenting patients with VBAO.

Our findings suggest that the late-presenting patients with VBAO may still benefit from EVT, underscoring the urgent need for RCTs to validate the efficacy and safety of EVT in this very late time window. The ongoing Efficacy and Safety of Endovascular Recanalization for Acute Basilar Artery Occlusion With Extended Time Window (ANGEL-BAO) RCT (NCT06101667) will provide further evidence for this population.

### Limitations

Our study has several limitations. First, it has some limitations that are inherent to observational registry studies: Selection bias may have occurred, favoring the inclusion of patients with high median PC-ASPECTS and narrow IQRs, as well as a preference for treating patients with EVT if they had isolated VBAO. Baseline differences persisted in some variables despite rigorous adjustment using PSM and IPTW. Residual or unmeasured confounding may remain, as with all observational studies. Second, the generalizability of our findings to other populations may be limited given that the study was conducted exclusively in China, where large artery atherosclerosis is a predominant stroke etiology. Third, the lack of serial NIHSS score documentation precluded assessment of dynamic neurological changes or disease progression. This is particularly relevant for patients with a symptom onset later than 24 hours given that early neurological fluctuations may not have been captured. Fourth, while structured telephone interviews improved follow-up completeness and quality control measures enhanced data reliability, reliance on telephone-based outcomes for a large proportion of patients may have still introduced measurement bias.

## Conclusions

In this cohort study, we found that selected patients with VBAO who received EVT beyond 24 hours after symptom onset had a greater increase in the likelihood of good functional status at 3 months compared with those who received BMT. The potential benefit associated with EVT may be greater in patients with baseline NIHSS scores of 10 or greater. However, more rigorous data from RCTs are needed to definitively establish the role of EVT in this patient population.

## Notes

Supplement 1. **eFigure.** Study flowchart **eTable 1.** Adjusted association of effectiveness and safety outcomes with EVT **eTable 2.** *E*-value for variable (SMD＞0.1 to <0.2) after PSM

Supplement 2.
Data Sharing Statement
