# Supplementary material for: Outcomes of Endovascular Treatment in Patients With Vertebrobasilar Artery Occlusion Beyond 24 Hours
Source: JAMA Netw Open. 2025 Jun 13;8(6):e2515526. doi: 10.1001/jamanetworkopen.2025.15526 (PMC12166486; doi:10.1001/jamanetworkopen.2025.15526)
Supplement: Supplement 1. — eFigure. Study flowchart eTable 1. Adjusted association of effectiveness and safety outcomes with EVT eTable 2. E-value for variable (SMD＞0.1 to <0.2) after PSM [file jamanetwopen-e2515526-s001.pdf]

Supplemental Online Content

Liu S, Xu Y, Nguyen TN, et al. Outcomes of endovascular treatment in patients with vertebrobasilar artery occlusion beyond 24 hours. *JAMA Netw Open*. 2025;8(6):e2515526. doi:10.1001/jamanetworkopen.2025.15526

eFigure. Study flowchart

eTable 1. Adjusted association of effectiveness and safety outcomes with EVT

eTable 2. *E*-value for variable (SMD＞0.1 to <0.2) after PSM

This supplemental material has been provided by the authors to give readers additional information about their work.

© 2025 Liu S et al. *JAMA Network Open*.

eFigure. Study flowchart

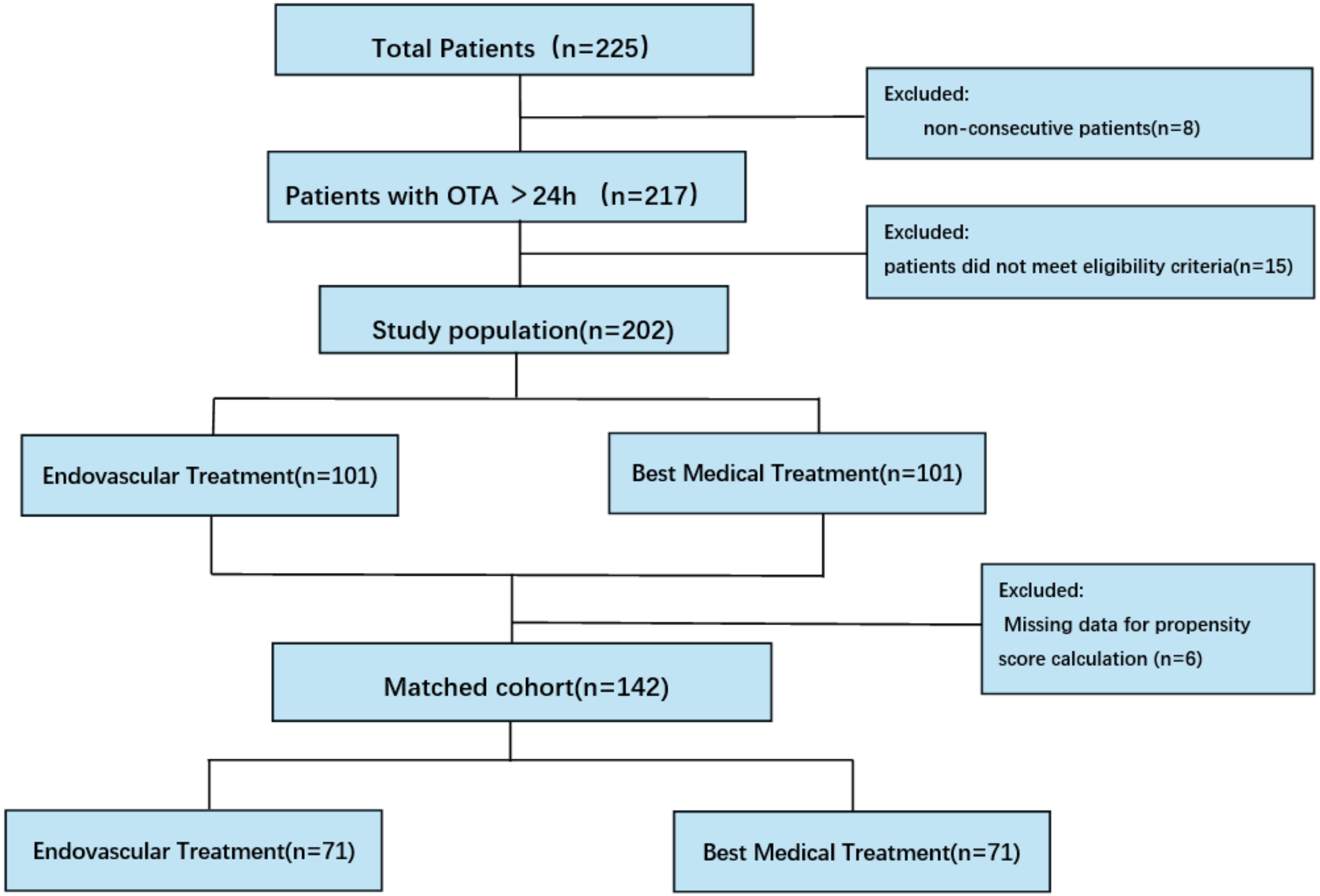

© 2025 Liu S et al. *JAMA Network Open*.

This figure shows the enrollment information of patients in the present study.

Abbreviations: OTA: estimated onset time from basilar artery occlusion to admission.

© 2025 Liu S et al. *JAMA Network Open*.

eTable 1. Adjusted association of effectiveness and safety outcomes with EVT

		Unmatch cohort	PSM cohort	IPTW cohort
**Primary Outcome**	**Measure of effect**	**Adjusted Value (95% CI)**	**Adjusted Value (95% CI)**	**Adjusted Value (95% CI)**
mRS 0-3 at 90d	Adjusted Risk Ratio	1.18(0.92,1.50)	1.35(1.02,1.79)	1.33(1.04,1.71)
**Secondary clinical outcomes**				
mRS at 90d	Common odds ratio	1.41(0.84,2.39)	1.54(0.85,2.81)	1.45(1.01,2.11)
mRS 0-1 at 90d	Adjusted Risk Ratio	0.68(0.43,1.10)	0.77(0.45,1.35)	0.82(0.52,1.29)
mRS 0-2 at 90d	Adjusted Risk Ratio	1.16(0.84,1.59)	1.29(0.90,1.85)	1.34(0.99,1.81)
mRS 0-4 at 90d	Adjusted Risk Ratio	1.21(1.01,1.47)	1.33(1.08,1.65)	1.35(1.12,1.64)

© 2025 Liu S et al. *JAMA Network Open*.

NIHSS score at 5-7 or discharge	Adjusted Mean Difference	-4.65(-7.34,-1.97)	-4.39(-7.40,-1.40)	-4.7(-7.03,-2.38)
**Safety outcomes**				
mortality at 90d	Adjusted Risk Ratio	0.92(0.84,1.00)	0.87(0.79,0.96)	0.57(0.34,0.96)
sICH at 3d	p value	(5.94%VS0.00%)0.038	(5.63%VS0.00%)0.128	(5.63%VS0.00%)0.128

Abbreviations: NIHSS=National Institutes of Health Stroke Scale; PSM=Propensity Score Matching; IPTW=Inverse Probability of Treatment Weighting; mRS=modified Rankin Scale; sICH=symptomatic intracranial hemorrhage; BMT=best medical treatment; EVT=endovascular treatment; CI=confidence interval.

© 2025 Liu S et al. *JAMA Network Open*.

eTable 2. E-value for Variable (SMD＞0.1 but <0.2) after PSM

Variable	SMD	E-value
Group（EVT）		3.76
Diabetes	11.7	3.29
Coronary heart disease	11.1	3.71
Anticoagulant	19.2	2.82
Antiplatelet	19.8	3.17
estimated time from basilar artery occlusion to admission	15.1	3.24

© 2025 Liu S et al. *JAMA Network Open*.

Baseline MRI pc-ASPECTS	14.4	3.33
Baseline MRI-PMI	14	3.01
Occlusion site	11.3	3.31

© 2025 Liu S et al. *JAMA Network Open*.
