# Supplementary material for: Outcomes of Endovascular Treatment in Patients With Vertebrobasilar Artery Occlusion Beyond 24 Hours
Source: JAMA Netw Open. 2025 Jun 13;8(6):e2515526. doi: 10.1001/jamanetworkopen.2025.15526 (PMC12166486; doi:10.1001/jamanetworkopen.2025.15526)
Supplement: Supplement 2. — Data Sharing Statement [file jamanetwopen-e2515526-s002.pdf]

Data Sharing Statement

Liu. Outcomes of Endovascular Treatment in Patients With Vertebrobasilar Artery Occlusion Beyond 24 Hours. *JAMA Netw Open*. Published online June 13, 2025. doi:10.1001/jamanetworkopen.2025.15526

Data
Data available: No
